# Song audio features are associated with the presence of alcohol references in popular music lyrics

**DOI:** 10.1093/alcalc/agag041

**Published:** 2026-06-11

**Authors:** Gedefaw D Alen, Emmanuel Kuntsche, Dan Anderson-Luxford, Zhen He, Benjamin Riordan

**Affiliations:** Centre for Alcohol Policy Research, Building NR1, La Trobe University, Plenty Road, Bundoora, VIC, 3086, Australia; Department of Public Health, College of Medicine and Health Science, Debre Markos University, Haddis Alemayehu Road, Gebeya, Debre Markos, Amhara, 073, Ethiopia; Centre for Alcohol Policy Research, Building NR1, La Trobe University, Plenty Road, Bundoora, VIC, 3086, Australia; Centre for Alcohol Policy Research, Building NR1, La Trobe University, Plenty Road, Bundoora, VIC, 3086, Australia; Department of Computer Science and Information Technology, GS2, La Trobe University, Plenty Road, Bundoora, VIC, 3086, Australia; Centre for Alcohol Policy Research, Building NR1, La Trobe University, Plenty Road, Bundoora, VIC, 3086, Australia

**Keywords:** alcohol reference, audio features, media exposure, popular music

## Abstract

**Aims:**

Preliminary evidence suggests that exposure to alcohol-related content in popular music is associated with drinking behaviour, especially amongst young people. Guided by Uses and Gratifications Theory, which posits that audiences select music to satisfy psychological and social needs, alcohol references, and specific audio features may co-occur as part of shared emotional and social experiences. This study assessed whether song audio features are associated with alcohol references in lyrics and whether they could support population-level surveillance of alcohol-related media exposure.

**Methods:**

We analyzed 6110 Billboard Top 100 songs released between 1959 and 2020, extracting Spotify audio features for each track. Logistic regression models were used to examine associations between audio features and the presence of alcohol references in lyrics.

**Results:**

Alcohol references were found in 16.1% of songs, with prevalence increasing significantly over time. Songs with higher danceability (OR = 1.35, *P* < .001), speechiness (OR = 1.39, *P* < .001), liveness (OR = 1.09, *P* = .021), positive valence (OR = 1.11, *P* = .041) and major mode (OR = 1.18, *P* = .042) more likely to contain alcohol references. Higher acousticness (OR = 0.82, *P* < .001) and instrumentalness (OR = 0.85, *P* = .015) were associated with lower odds. Loudness, tempo, and energy were not significantly associated.

**Conclusions:**

Audio features may help identify songs more likely to contain alcohol references in music and may have potential utility for monitoring alcohol-related messaging across large volumes of popular music. Such tools could complement broader public health efforts to reduce alcohol exposure, especially amongst young people.

## Teaser

**Short Summary** Songs in the Billboard top 100 that had higher danceability, liveness, and speechiness were more likely to reference alcohol. In contrast, songs that were higher in acousticness and instrumentalness was less likely to reference alcohol, whilst loudness and energy showed no significant association.

## Introduction

The rise of smart devices and streaming platforms has led to increased global music consumption, with weekly listening time increasing from 18.4 h in 2021 to 20.7 h in 2023 (International Federation of the Phonographic Industry (IFPI) 2023). Although music is associated with a range of positive outcomes, this rise in music consumption raises concerns about exposure to content that may negatively impact health behaviours, such as exposure to content promoting alcohol consumption. Indeed, previous studies found that there is a positive link between exposure to alcohol-related content in popular songs and alcohol drinking behaviours (Engels *et al*. 2011, Herd 2014, Primack *et al*. 2014).

Prior research has reported a high prevalence of alcohol references and alcohol-related themes in song lyrics (Hall *et al*. 2013, Hardcastle *et al*. 2013, Herd 2014, Pettigrew *et al*. 2018, Christenson *et al*. 2019). Notably, a recent meta-analysis of 23 studies reported that approximately one in five popular songs (22.0%) referenced alcohol in their lyrics (Alen *et al*. 2024). Interestingly, evidence suggests that alcohol references have become more common in popular music and have increased overtime (Pettigrew *et al*. 2018). For example, in a content analysis of Billboard Hot 100 year-end songs (1959–2009), songs released in 2009 had ~18 times the odds of containing alcohol, tobacco, or other drug references compared with songs from 1959, and alcohol accounted for the large majority of substance references overall (88.8%) (Hall *et al*. 2013). Similarly, in a longitudinal analysis of UK charting songs, the prevalence of alcohol references increased markedly over time, rising from 2.1% in 1991 to 8.1% in 2001 and 18.5% in 2011 (Hardcastle *et al*. 2015).

Beyond year, most studies to date have relied on genre-based comparisons of the prevalence of alcohol references (Primack *et al*. 2008, Siegel *et al*. 2013, Holody *et al*. 2016). These studies suggest that certain genres (e.g. rap/hip-hop), are more likely to feature alcohol-related content (Primack *et al*. 2008, Siegel *et al*. 2013, Holody *et al*. 2016). For example, Primack *et al*. (2008) analyzed 279 Billboard songs and found that alcohol references were most prevalent in rap songs (53%) compared with pop (6%) and rock (6%). Although genre can potentially be helpful for estimating alcohol references, genre classification can be subjective, and often difficult to determine consistently across sources. In addition, musicians blend different stylistic elements when making music and the genre of music may differ substantially even within an artist’s own catalogue of music, for songs on the same album, and even within the same song. Furthermore, genre is difficult to consistently estimate, and Spotify’s genre system includes thousands of genres (Krogh 2025) and different sources may classify the same song differently (e.g. Taylor Swift’s ‘You Belong with Me’ is listed as ‘pop’ on Spotify, but ‘country’ on last FM). Genre themselves itself can even change over time, for example, songs that were considered progressive or metal are today considered classic rock. Given the difficulties in estimating genre, objectively measured audio features may offer a more detailed and reproducible way to characterize songs that reference alcohol. For example, measures like beats per minute (BPM), danceability, energy, valence, and speechiness of a song can be estimated objectively using algorithms. Although genre may partly reflect some of these features (e.g. dance music has a higher BPM), songs within these genres may differ on other characteristics that may be more informative for predicting whether alcohol is referenced (e.g. valence). Thus, beyond genre, analyzing the relationship between a song’s audio features and its lyrical content may allow us to identify songs more likely to contain alcohol-related themes.

Guided by Uses and Gratifications Theory, which posits that audiences actively select media to satisfy psychological and social needs (e.g. mood regulation, arousal, and social connection), we propose that alcohol references in lyrics will be more common in songs with particular audio features (Ruggiero 2000, Pridy *et al*., 2021). Musical audio features, such as tempo (BPM), key/mode, danceability, energy, and valence, shape the emotional tone of a track and help listeners achieve these gratifications (e.g. high BPM/energy and high danceability for celebration and social bonding; slower tempos, minor mode, and lower valence for reflection or coping) (Ilie and Thompson 2006, Gabrielsson and Lindström 2010, Panda *et al*. 2023). Similarly, alcohol references can function as culturally shared symbols that further specify the intended context and emotion, signalling partying and sociability in upbeat tracks (e.g. ‘popping champagne’) or coping and escape in lower-valence songs (e.g. drinking to ‘numb the pain’). Thus, specific audio features and alcohol-related lyrics may co-occur because they reflect shared gratification targets rather than a direct causal relationship. Artists and producers may co-select specific audio features and alcohol-related lyrics to create cohesive listening experiences suited to anticipated situations (e.g. clubs, workouts, breakups), and these combinations may become more common over time through listener preferences, genre conventions, and platform-driven exposure. Accordingly, it is plausible that audio features may be associated with the presence of alcohol references, and this warrants further investigation.

In recent years, Spotify, the most popular music streaming platform, provides access to song-level audio features through its Application Programming Interface (Spotify. 2025), enabling detailed analysis of how measurable song characteristics relate to lyrical content and listening contexts. For example, a large-scale study of Spotify playlists labelled for studying or sleeping across multiple genres found that music intended for studying and sleeping typically had lower valence, energy, tempo, and loudness, and higher acousticness and instrumentalness compared with general music playlists (Scarratt *et al*. 2023). Additionally, a study on daily music preferences found that audio features vary throughout the day, with morning music favouring higher loudness, energy, and valence, whilst evening preferences shift towards quieter, slower-tempo songs for relaxation (Heggli *et al*. 2021). These findings suggest that audio features can reflect the functional context of songs, raising the possibility that certain features may also be associated with lyrical references to alcohol-related themes.

Whilst lyric analysis remains the most widely used approach for detecting alcohol in songs, it is often time-consuming and labour-intensive (Alen *et al*. 2024, Tran *et al*., 2024, Herd 2014). Given audio features have been shown to reliably predict specific functional and contextual aspects of music listening (Heggli *et al*. 2021, Scarratt *et al*. 2023), it is important to extend understandings of the relationship between audio features and alcohol references. This is particularly relevant given substantial changes in music styles and cultural attitudes over time, which may have altered both the audio characteristics of songs, and the ways alcohol is portrayed. If track-level audio features are associated with alcohol references in lyrics, they may offer a useful first step for flagging songs more likely to contain such content. These songs could then be prioritized for further review using lyric-based artificial intelligence methods, such as the Lyrics-based Deep Learning Algorithm (LYDIA), to confirm alcohol-related references (Bonela *et al*. 2024). This approach could support public health surveillance of alcohol-related themes in popular music and inform media literacy efforts aimed at reducing youth exposure. Therefore, this study primarily aimed to assess the relationship between Spotify audio features and alcohol references in song lyrics. Given that music styles and cultural attitudes towards alcohol have evolved over time, we also explored whether these associations have changed across time by examining interactions between audio features and time (in decades) in predicting alcohol references.

## Methods

### Study sample and data collection procedures

A total of 6110 songs from the Billboard Top 100 charts between 1959 and 2020 were retrieved. Song titles and artist names were identified using Wikipedia, and lyrics for eligible songs were obtained via the Genius API. Audio features for each song were retrieved from Spotify’s Application Programming Interface (API).

The presence of alcohol-related content in song lyrics was determined using a compiled list of 673 alcohol-related keywords. Two researchers independently reviewed the lyrics, with any discrepancies resolved through discussion. Further details on data collection and processing are available in a previously published paper (Bonela *et al*. 2024).

#### Definitions of alcohol reference and Spotify audio features

Songs were classified as referencing alcohol if they contained at least one alcohol-related reference in the lyrics. This included both explicit references (e.g. mentions of alcohol, alcoholic beverages, intoxication, brand names, or drinking-related locations such as bars or pubs) and implicit references, where the context or situation suggested alcohol use without direct mention (e.g. ‘pour another round,’ ‘shots on me,’ or ‘raise a glass’).

Spotify provides a range of audio features for each song. The audio features analyzed in this study include danceability (suitability for dancing), energy (intensity of the music), acousticness (likelihood of being acoustic), instrumentalness (absence of vocals), liveness (presence of a live audience), speechiness (number of spoken words), and valence (positivity or emotional tone), each scored from 0.0 to 1.0, with higher values indicating a stronger presence of that characteristic. In addition, tempo was measured in beats per minute, loudness was measured in decibels, and mode was coded as a binary variable indicating whether the track was in a major or minor key.

### Statistical analysis

We used mean values with standard deviation to summarize quantitatively measured characteristics of songs. We examined temporal trends in audio features over time (1959–2020) using regression models with year as the time variable, and visualized trends using mean values of the audio features over time. We also calculated decade-specific proportions of songs containing alcohol-related content, separately for explicit and implicit references, and displayed the results graphically.

To examine the relationship between song audio features and the presence of alcohol references in lyrics, we fitted a logistic regression model. Continuous audio features were standardized (mean = 0, SD = 1) to facilitate comparability of effect sizes across features and improve model convergence. Decade of release was included as a fixed effect to account for shared cultural and musical context amongst songs released within the same decade. Decade was treated as a proxy for broad, unmeasured contextual influences (e.g. shifts in cultural norms and industry practices) rather than as a specific explanatory variable. Songs were grouped by decade rather than by year to capture broader temporal trends and improve model stability. Because 1959 and 2020 each included only 100 songs, these years were grouped with the nearest decade for the regression models. We then tested whether associations between song characteristics and alcohol references changed over time by including interaction terms between audio features and decade, using the 1960s as the reference decade.

The strength of associations was reported as odds ratios (OR) with 95% confidence intervals (CI). Because continuous audio features were standardized before analysis, ORs represent the change in the odds of an alcohol reference associated with a one-standard-deviation increase in each feature. All analyses were performed using R (version 4.3.3).

## Results

### Change in song audio features over time

**Figure 1:**
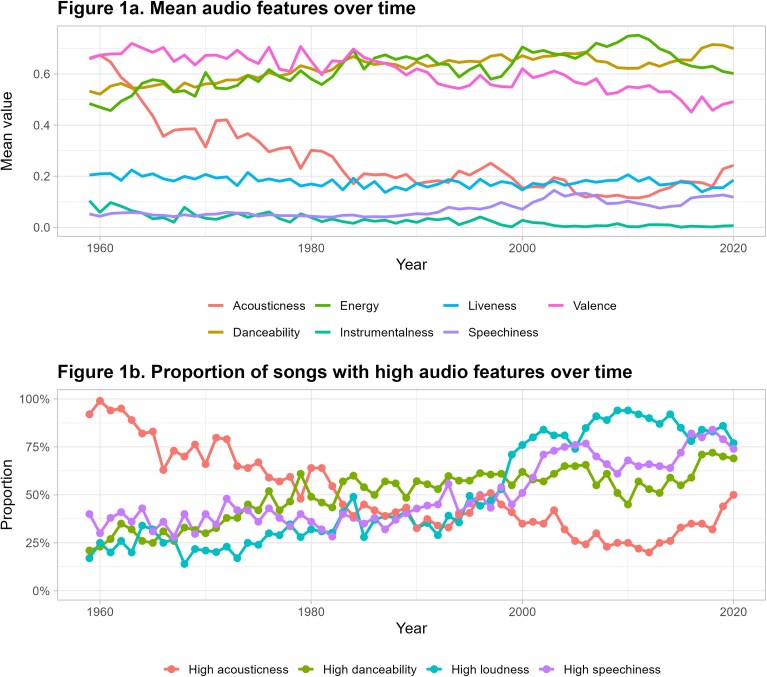
Change in song audio features over time.

Analysis of the mean values of key audio features showed significant changes in the characteristics of popular music over time (1959–2020; *P* < .001). As shown in Fig. 1a, danceability, energy, and speechiness steadily increased, indicating that songs have become more rhythmic, energetic, and have more spoken words. In contrast, acousticness and valence consistently declined over time. These trends highlight a clear change in the audio characteristics of Billboard Top 100 songs across decades (Fig. 1a).

To further explore the trends observed in Fig. 1a and b shows the yearly proportion of Billboard Top 100 songs classified as having high levels of danceability, loudness, speechiness, and acousticness. The results showed that the proportion of songs with higher values for these audio features has changed over time within the annual Billboard charts.

### Proportion of songs with alcohol references

In this study, of the 6110 songs, 16.1% (n = 984) contained at least one alcohol-related reference, whilst 11.3% explicitly mentioned alcohol by directly using terms such as ‘beer’, ‘wine’, or ‘whiskey’. Amongst the songs that referenced alcohol, 56% referenced alcohol two or more times in the lyric text as provided (including repeated chorus lines where applicable).

**Figure 2:**
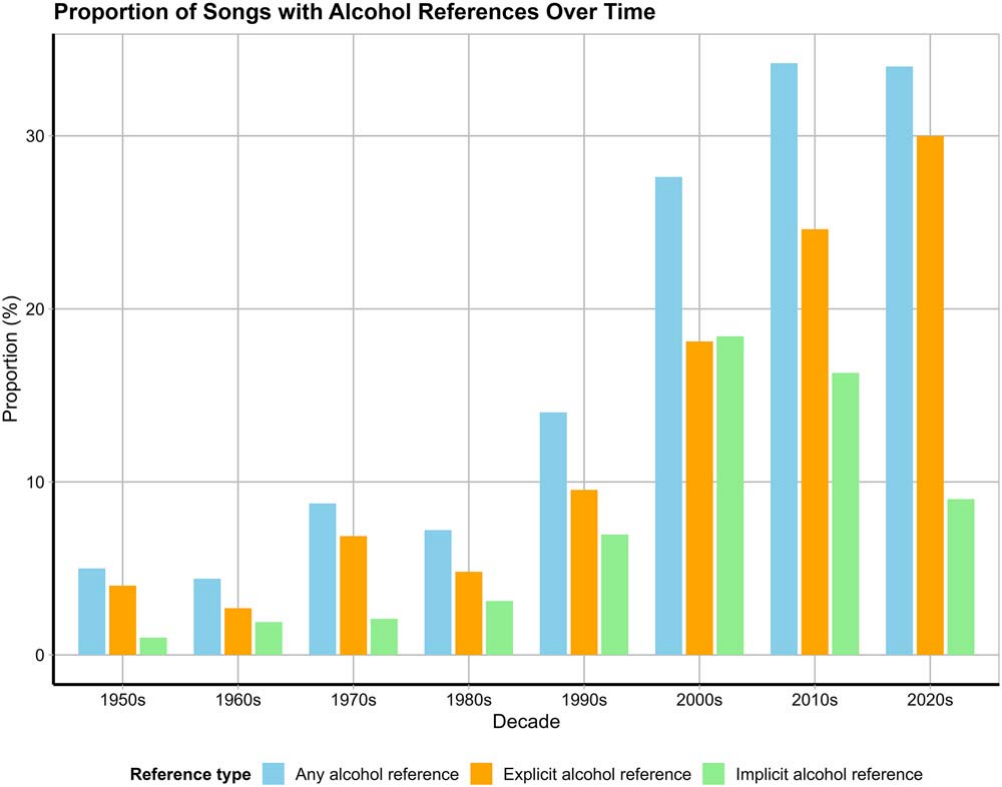
Proportion of songs with alcohol references per decade.

The proportion of Billboard’s Top 100 songs referencing alcohol increased over time. As shown in Fig. 2, from the 1950s to the 1980s, alcohol references remained relatively stable, fluctuating between 5.0% and 9.0%. This prevalence increased in the 1990s to 14.0%, and sharply increased thereafter, reaching ~35.0% in the 2010s and 2020s. (the yellow-coloured bar) also shows an increase in the proportion of songs with explicit alcohol references between the 2010s and 2020s. *Note: The data for the 1950s and 2020s reflect only the years 1959 and 2020, respectively (n = 100 songs each), as complete decade-level data were not available.*

### The relationship between song features and alcohol reference

As shown in Table 1, in multivariable logistic regression adjusting for decade, several audio features were significantly associated with the presence of alcohol references in lyrics. Specifically, higher danceability (OR = 1.35, 95% CI:1.22–1.49), higher speechiness (OR = 1.39, 95% CI: 1.30–1.48), and higher liveness (OR = 1.09, 95% CI:1.01–1.18) were significantly associated with higher odds of alcohol references. In contrast, higher acousticness (OR = 0.82, 95% CI: 0.73–0.91) and higher instrumentalness (OR = 0.85, 95% CI: 0.75–0.97) were associated with lower odds of alcohol reference.

**Table 1:** The relationship between song features and alcohol references.

**Variable**	**Reference any alcohol related contents (Yes vs No)**	
	**OR (95% CI)**	***P*-value**
**Level 1 variables (song features)**		
Danceability	1.35(1.22–1.49),	< .001
Valence	1.11(1.00–1.22)	.041
Energy	1.01(0.88–1.15)	.915
Speechiness	1.39(1.30–1.48)	< .001
Acousticness	0.82(0.73–0.91)	< .001
Liveness	1.09(1.01–1.18)	.021
Instrumentalness	0.85(0.75–0.97)	.015
Tempo	0.97(0.90–1.05)	.454
Loudness	0.93(0.81–1.06)	.271
Mode (major vs minor)	1.18(1.01–1.39)	.042
**Decade**		
1960s	Ref.	Ref.
1970s	1.71(1.18–2.48)	.005
1980s	1.25(0.84–1.86)	.261
1990s	2.20(1.51–3.20)	< .001
2000s	4.51(3.09–6.60)	< .001
2010s	6.96(4.76–10.18)	< .001
**Model fit parameter**		
Pseudo R^2^	.278	

OR = odds ratio; CI = confidence interval. OR Values > 1 indicate higher odds of alcohol-related content. Ref. = Reference.

Valence and mode showed modest evidence of association. Higher valence was associated with slightly higher odds of alcohol references (OR = 1.11, 95% CI: 1.00–1.22), whilst songs in a major mode had slightly higher odds of alcohol references than songs in a minor mode (OR = 1.18, 95% CI: 1.01–1.39). In contrast, energy, tempo, and loudness were not clearly significantly associated with alcohol references (Table 1).

Similarly, we observed a clear temporal increase in alcohol references across decades. Compared to the 1960s, the odds of alcohol references were higher in the 1970s (OR = 1.71, 95% CI 1.18–2.48), and more than doubled in the 1990s (OR = 2.20, 95% CI: 1.51–3.20). The association was stronger in later decades, with songs from the 2000s shower over fourfold higher odds (OR = 4.51, 95% CI: 3.09–6.60) and songs from the 2010s showing nearly sevenfold higher odds (OR = 6.96, 95% CI: 4.76–10.18) of referencing alcohol as compared with songs from the 1960s (*P* < .001). No significant differences were observed for the 1980s. These results highlight a marked rise in alcohol references beginning in the 1990s (Table 1).

### Temporal variation in the relationship between song features and alcohol references

**Figure 3:**
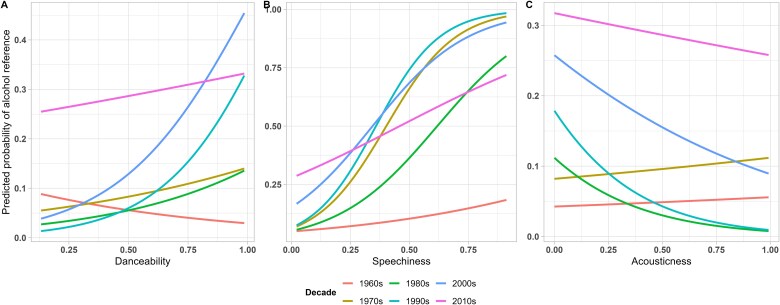
Interaction plot between danceability, speechiness, acousticness, and time (decade) on alcohol references.

The cross-level interaction analysis results revealed that the relationship between song audio features and alcohol references has changed over time. As shown in Fig. 3, the relationship between certain song features (danceability and speechiness) and alcohol references has become more pronounced in recent decades, particularly in the 1990s and 2000s, compared to earlier decades. In the 1990s and 2000s, songs with higher danceability and speechiness were significantly more likely to reference alcohol. In contrast, earlier decades showed weaker or even opposite trends. For example, in the 1960s, more danceable songs had fewer alcohol references. Similarly, acoustic songs were linked to more alcohol content in early decades but less in later years.

## Discussion

This study investigated the relationship between Spotify audio features and alcohol references in song lyrics over more than six decades. We found that songs with higher danceability, high speechiness, high liveness, positive valence, major mode, and lower acousticness had higher odds of referencing alcohol. Importantly, the relationship between these audio features and alcohol references varied across time periods, suggesting that the musical characteristics associated with alcohol-related content have changed over time.

These findings can be interpreted through the lens of Uses and Gratifications Theory, which suggests that media content is shaped by, and responds to, users’ psychological and social needs. Alcohol references were more likely to appear in songs with features associated with celebratory, socially engaging contexts, such as higher danceability, liveness, and positive valence. This may indicate that specific audio features and alcohol references co-occur because they align with psychological and social needs, such as mood regulation, arousal, and social connection (Ruggiero 2000, Pridy *et al*., 2021). Specifically, more rhythmic and speech-oriented songs may be more likely to include alcohol-related themes, perhaps because such songs are more readily selected for party and other social settings in which alcohol is commonly reference. This is consistent with previous research indicating that genres such as R&B and hip-hop, which are often characterized by high danceability and speechiness, tend to contain a higher prevalence of alcohol references (Siegel *et al*. 2013, Holody *et al*. 2016). However, because track-level genre data were unavailable and could not be included in the current analysis, the extent to which these associations reflect genre differences cannot be fully determined. Therefore, these findings should be interpreted with caution.

Despite these associations, audio features explained only part of the variation in alcohol references (Pseudo R^2^ = 0.278), highlighting the heterogeneity of alcohol-related content in music. Alcohol references are not confined to a single set of musical characteristics but instead appear across a wide range of contexts, including celebratory, social, commercial, emotional, and coping-related themes. Consistent with this, prior studies have shown that alcohol portrayals vary across genres and narratives, ranging from associations with partying and wealth to themes of loneliness, loss, and negative consequences, particularly in genres such as country music (Primack *et al*. 2008, Siegel *et al*. 2013, Holody *et al*. 2016).

Taken together, these findings suggest that whilst certain audio features are associated with a higher likelihood of alcohol references, these features are unlikely to replace lyrics analysis given the observed heterogeneity and substantial unexplained variation. Rather, as standardized, reproducible, and fine-grained track-level measures of musical characteristics (Spotify. 2025), audio features may help identify broad patterns in alcohol-related music content or flag songs with a higher likelihood of containing alcohol references, particularly when used alongside lyric-based content analysis. In practice, these songs could then be prioritized for more detailed lyric-based or artificial intelligence-assisted analysis. This approach may support large-scale monitoring of alcohol-related content in music, inform media literacy initiatives by identifying music environments where alcohol references are more common, and assist parents and caregivers in recognizing higher-risk content. From a public health perspective, these findings may help inform broader preventive measures to reduce alcohol exposure amongst at-risk populations, including platform-level monitoring of music content, clearer labelling of alcohol-related material, age-appropriate classification systems, and media literacy strategies. In this context, audio features may serve as a complementary signal for identifying songs with a higher likelihood of alcohol-related content in large music datasets.

The findings of this study also showed broader temporal changes in the musical characteristics of popular songs. Specifically, danceability, energy, and speechiness of songs increased across the 62-year period, whilst acousticness and valence declined over time. These changes suggest a shift in the overall audio characteristics of Billboard Top 100 songs, becoming more upbeat, dynamic, and rhythm-driven, with more softened acoustic and less emotional tones. The declining trend in valence aligns with findings by Dodds *et al*., who reported a decline in the happiness levels of song (i.e. valence) lyrics over time (Dodds and Danforth 2010).

Another key finding of the current study was that the relationship between song audio features and alcohol references changed significantly over time. Specifically, the stronger positive association between danceability and alcohol references in the 1990s and 2000s, compared to the opposite pattern observed in the 1960s, may reflect broader shifts in social norms, the rise of nightlife and club culture, and the increasing commercialization of alcohol in entertainment settings (Herd 2014). Additionally, the significant effect of speechiness in the 1990s likely reflects the growing prominence of hip-hop, a genre known for spoken-word lyrics and themes related to partying (Blair 1993, Stickle 2021). These temporal patterns may also suggest a process of norm formation and reinforcement. Repeated pairing of alcohol references with energetic and socially oriented musical features may strengthen expectations that alcohol is part of these contexts and, over time, may contribute to the normalization of alcohol within upbeat and party-oriented music environment (Hawkins *et al*. 2019). Importantly, the alcohol reference itself may not be the only relevant cue, because energetic, rhythmic, and speech-oriented songs may more broadly evoke drinking-related contexts, making musical style a possible signal for alcohol-related expectations and behaviours.

Consistent with previous studies (Herd 2005, Hardcastle *et al*. 2015, Pettigrew *et al*. 2018), the current study also showed that the prevalence of alcohol references in popular music increased over time, rising from 5.0% in the 1960s to 35% in the 2010s. This pattern may reflects broader societal changes, including shifting cultural attitudes towards alcohol (Chang *et al*. 2008), the commercialization of alcohol in media and the growing normalization of alcohol-related behaviour in popular culture (Carah and Brodmerkel 2021, Stickle 2021). This concerning because exposure to alcohol-related content in popular music may reinforce the perception that drinking is common and socially acceptable, particularly amongst younger audiences.

A key strength of this study is its large-scale analysis of Billboard Top 100 songs spanning over six decades (1959–2020). This broad scope overcomes a common limitation in previous research, which has often relied on smaller and genre-specific samples. By analyzing a large-scale and representative set of popular songs, our findings offer a more comprehensive view of how audio features and alcohol references have evolved over time. Additionally, this study is amongst the first to use Spotify’s audio features to examine their relationship with alcohol references, offering a novel and practical approach for identifying songs with a higher or lower likelihood of alcohol-related content.

Despite these strengths, several limitations should also be considered. First, our analysis includes only English-language Billboard songs, which may limit the generalizability of the findings to non-English music. Second, although Spotify-derived audio features provide scalable indicators of song characteristics, they do not capture all relevant musical characteristics or contextual factors. In particular, track-level genre information was not available in the Spotify audio-features dataset, which limited our ability to adjust for genre or conduct genre-stratified analyses. As a result, we could not determine whether the observed temporal trends reflect shifts in the genre composition of the Billboard Top 100 over time. Third, we were unable to incorporate detailed artist level characteristics (e.g. age, gender, race, background narratives) because these data were not available in the Spotify dataset. Finally, the reported associations may be influenced by unmeasured factors (e.g. broader changes in social norms, alcohol marketing, or industry practices) that could confound the relationships between audio features and alcohol references.

Future research should extend this work beyond English-language Billboard songs by examining non-English music and other charts or streaming-based rankings to assess generalizability across languages and cultural contexts. Studies that link songs to track-level genre metadata (e.g. via external databases or enriched platform metadata) are also needed to adjust for genre, conduct genre-stratified analyses, and determine whether observed temporal trends reflect shifts in the genre composition of popular music over time. In addition, integrating artist-level characteristics (e.g. branding, and collaboration networks, and where appropriate ethically sourced demographic attributes) would help clarify how artist level factors shape both musical style and alcohol-related lyrical content. Finally, incorporating direct measures of social and industry context, such as alcohol marketing, sponsorship trends, and relevant policy or regulatory changes, would reduce unmeasured confounding and strengthen interpretation of observed associations.

## Conclusion

Specific audio features were significantly associated with the presence of alcohol references in popular music. Song features such as danceability, speechiness, liveness, and acousticness may serve as useful indicators of alcohol-related content, offering a practical and scalable approach for classifying and monitoring alcohol exposure in music, particularly amongst young audiences. These can inform content warning systems and music classification tools; support media literacy programmes by helping educators and parents identify songs with higher-risk content; and help public health efforts to monitor and reduce youth exposure to alcohol promotion through music. Future research should investigate the predictive power of audio features for identifying alcohol references in music, using advanced models like neural networks or other machine learning models.

## Data Availability

The song metadata analysed in this study were obtained from publicly accessible sources, specifically the Spotify Web API. Aggregated data supporting the findings of this study are available from the corresponding author upon reasonable request.
